# Ethanolic Extract of *Polygonum minus* Protects Differentiated Human Neuroblastoma Cells (SH-SY5Y) against H_2_O_2_-Induced Oxidative Stress

**DOI:** 10.3390/molecules28186726

**Published:** 2023-09-20

**Authors:** Nor Hafiza Sayuti, Nabilah Zulkefli, Jen Kit Tan, Norazalina Saad, Syarul Nataqain Baharum, Hamizah Shahirah Hamezah, Hamidun Bunawan, Qamar Uddin Ahmed, Humaira Parveen, Sayeed Mukhtar, Meshari A. Alsharif, Murni Nazira Sarian

**Affiliations:** 1 Institute of Systems Biology (INBIOSIS), Universiti Kebangsaan Malaysia, Bangi 43600, Malaysia; norhafizasayuti@ukm.edu.my (N.H.S.); p115964@siswa.ukm.edu.my (N.Z.); nataqain@ukm.edu.my (S.N.B.); hamizahshahirah@ukm.edu.my (H.S.H.); hamidun.bunawan@ukm.edu.my (H.B.); 2 Department of Biochemistry, Faculty of Medicine, Universiti Kebangsaan Malaysia, Bandar Tun Razak, Cheras, Kuala Lumpur 56000, Malaysia; 3 UPM-MAKNA Cancer Research Laboratory, Institute of Bioscience, Universiti Putra Malaysia, Serdang 43400, Malaysia; norazalina@upm.edu.my; 4 Drug Discovery and Synthetic Chemistry Research Group, Department of Pharmaceutical Chemistry, Kulliyyah of Pharmacy, International Islamic University Malaysia, Kuantan 25200, Malaysia; quahmed@iium.edu.my; 5 Department of Chemistry, Faculty of Science, University of Tabuk, Tabuk 71491, Saudi Arabia; h.nabi@ut.edu.sa (H.P.); snoor@ut.edu.sa (S.M.); 6 Department of Chemistry, Faculty of Applied Sciences, Umm Al-Qura University, Makkah 21955, Saudi Arabia; maasharif@uqu.edu.sa

**Keywords:** *Polygonum minus*, oxidative stress, antioxidant, neuron, neuroprotective, hydrogen peroxide, acetylcholine, molecular docking, LC–MS/MS

## Abstract

Neuronal models are an important tool in neuroscientific research. Hydrogen peroxide (H_2_O_2_), a major risk factor of neuronal oxidative stress, initiates a cascade of neuronal cell death. *Polygonum minus* Huds, known as ‘kesum’, is widely used in traditional medicine. *P. minus* has been reported to exhibit a few medicinal and pharmacological properties. The current study aimed to investigate the neuroprotective effects of *P. minus* ethanolic extract (PMEE) on H_2_O_2_-induced neurotoxicity in SH-SY5Y cells. LC–MS/MS revealed the presence of 28 metabolites in PMEE. Our study showed that the PMEE provided neuroprotection against H_2_O_2_-induced oxidative stress by activating the Nrf2/ARE, NF-κB/IκB and MAPK signaling pathways in PMEE pre-treated differentiated SH-SY5Y cells. Meanwhile, the acetylcholine (ACH) level was increased in the oxidative stress-induced treatment group after 4 h of exposure with H_2_O_2_. Molecular docking results with acetylcholinesterase (AChE) depicted that quercitrin showed the highest docking score at −9.5 kcal/mol followed by aloe-emodin, afzelin, and citreorosein at −9.4, −9.3 and −9.0 kcal/mol, respectively, compared to the other PMEE’s identified compounds, which show lower docking scores. The results indicate that PMEE has neuroprotective effects on SH-SY5Y neuroblastoma cells in vitro. In conclusion, PMEE may aid in reducing oxidative stress as a preventative therapy for neurodegenerative diseases.

## 1. Introduction

Oxidative stress is a condition that happens when there is an imbalance between oxidants and antioxidants in a living system. It is linked to many neurological disorders including Alzheimer’s disease, Huntington’s disease, Parkinson’s disease, and amyotrophic lateral sclerosis. The imbalance happens when there are too many reactive oxygen species (ROS) or when the antioxidant system does not work appropriately [1]. The body has specific defense mechanisms in the form of endogenous antioxidants, such as glutathione, and antioxidant enzymes, such as superoxide dismutase (SOD), glutathione peroxidase (GPX), and catalase, which combat the harmful effects of excessive ROS [2]. A growing body of research has revealed that, under normal conditions, oxidative damage causes reactions that have a negative impact on beneficial markers in the antioxidant mechanistic pathway that is responsible for neutralizing harmful stimuli. Exogenous antioxidants, on the other hand, tend to counteract such effects, but when present in large quantities, they inhibit the response generated by their indigenous counterpart, increasing the cells’ sensitivity to stimuli and eventually leading to death [3,4,5]. The antioxidant properties of plant materials for maintaining health and preventing various diseases prompted scientists to investigate various herbs.

*Polygonum minus* Huds is a well-known traditional herbal plant in Malaysia, and it is commonly referred to as ‘kesum’ in the Malay language [6]. Various Polygonum species have demonstrated antioxidant properties [7,8]. *P. minus* is a plant with a high concentration of flavonoids and other antioxidants that reduce oxidative stress in neuronal membranes [9]. *P. minus* has been shown to have in vitro antioxidant properties, low-density lipoprotein (LDL) oxidation inhibition, antiulcer activity, analgesic activity, anti-inflammatory activity, antiplatelet aggregation activity, antimicrobial activity, digestive enhancing property, and cytotoxic activity [10,11,12,13,14,15]. Researchers have linked this plant’s pharmacological effects to its high antioxidant capacity. This plant’s aqueous, methanolic, and ethanolic extracts demonstrated high antioxidant activity, which was primarily attributed to its phenolic compounds [10,16,17,18]. The isolated compounds from *P. minus* such as polygonumins A [19], polygonumins B, C, and D [6] were reported to have diverse potential medicinal activities. In a study conducted by Yaacob [20], it was found that decanal and dodecanal are the primary aldehydes responsible for contributing flavor to *P. minus*. In addition to decanal and dodecanal, Yaacob’s analysis revealed the presence of several other compounds in *P. minus*, including 1-decanol, 1-dodecanol, undecanal, tetradecanal, 1-undecanol, nonanal, 1-nonanol, and β-caryophyllene. In a study carried out by Baharum et al. [21], a total of 42 compounds were successfully identified using gas chromatography–mass spectrometry (GC–MS). This number greatly exceeded the count given by Yaacob [20]. Some of the compounds found included α-pinene, drimenol, humulene, caryophyllene, farnesol, neoisolongifolene, 8-bromo-, and isobornyl acetate. To date, the literature on the neuroprotective effect of *P. minus* is still lacking. Thus, this present study aims to determine whether *P. minus* ethanolic extract (PMEE) can protect differentiated human neuroblastoma SH-SY5Y cells from H_2_O_2_-mediated oxidative stress.

## 2. Results and Discussion

### 2.1. LC–MS/MS Analysis

**Figure 1:**
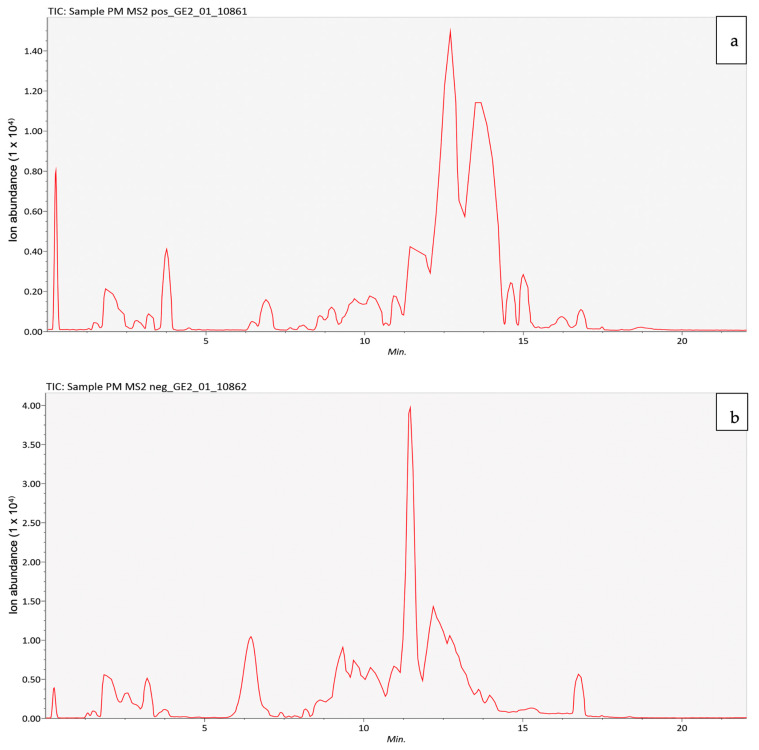
Liquid chromatography–mass spectrophotometry (LC–MS/MS) analysis of PMEE in the positive mode (**a**) and the negative mode (**b**).

The positive and negative LC–MS/MS chromatograms of PMEE were shown in Figure 1. The presence of peaks in the chromatogram indicated the presence of various PMEE-derived compounds. The LC–MS/MS characterization of the phenolic compounds in PMEE revealed the presence of 28 metabolites, listed in Table 1. These metabolites have a variety of therapeutic properties, including anti-inflammatory, antioxidant, and anticancer properties.

In Table 1, PMEE was shown to contain various classes of natural compounds such as caffeic acid, (−)-epicatechin, kaempferol, gallic acid, eupatilin (5,7-dihydroxy-3′,4′,6-trimethoxyflavone) and rhamnetin (7-methylquercetin) which have been proven to play a role in scavenging H_2_O_2_ and preventing cell damage by oxidative stress [22,23,24,25,26,27]. On the other hand, quercetin and quercitrin reduced the accumulation of ROS and nitric oxide while protecting against cytokine-induced cell death [28]. Meanwhile, Kwon et al. [29] demonstrated the neuroprotective activity of quinic acid isolated from the roots of *Arctium lappa* Linne. It protected PC12 cells from oxidative stress, which could be attributed to the antioxidant capacity of quinic acid [29]. The detection of various phenolic compounds, based on compound identification, strengthens the suggestion that other bioactive compounds in addition to polyphenols or flavonoids were also present and contributed to the diverse bioactive characteristics of *P. minus*.

### 2.2. Phase Contrast Microscopy and Immunocytochemistry Confirmed Neuronal Marker β-Tubulin III Expression

**Figure 2:**
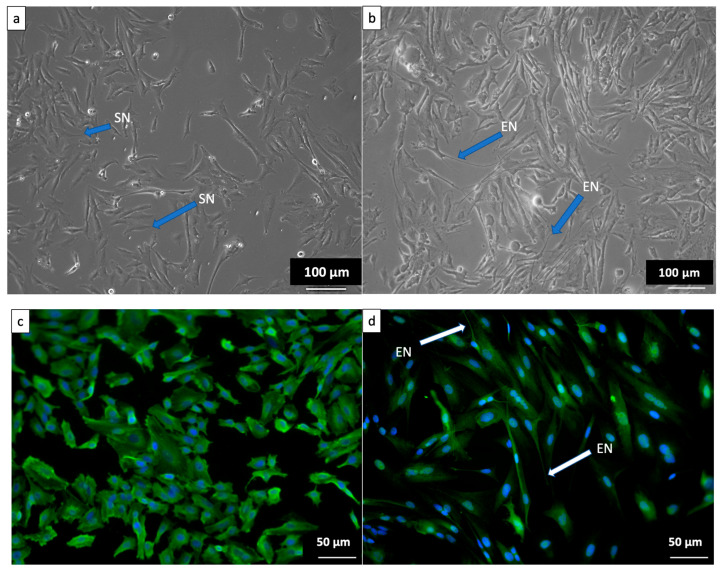
The image shows SH-SY5Y cells observed in phase contrast microscopy (**upper**) and differentiated SH-SY5Y cells observed in fluorescence microscopy (**lower**). (**a**) Undifferentiated SH-SY5Y cells cultured in complete growth media, (**b**) differentiated SHSY-5Y cells cultured in complete differentiation media treated with 10 μM retinoic acid (RA) for 7 days, (**c**) fluorescence micrograph of undifferentiated cells and the image (**d**) showing the extension of SH-SY5Y neurites after 7-day differentiation with 10 μM RA. SN: short neurites. EN: extended neurites. Magnification ((**a**,**b**): 10×; (**c**,**d**): 20×).

To demonstrate the neuronal phenotype of differentiated SH-SY5Y used in this study, the cells were examined under a fluorescence microscope using the phase contrast mode. Figure 2a shows that undifferentiated cells had no or significantly fewer neurites. In contrast, after seven days of differentiation, the neurite characteristics persisted and grew in the differentiated cells (Figure 2b), indicating that the SH-SY5Y cells had differentiated into typical neuronal cells. Immunochemistry was performed on differentiated cells in addition to morphological evaluation of SH-SY5Y-derived neuronal cells to evaluate the differentiation process. Undifferentiated and differentiated SH-SY5Y cells were stained and incubated with antibodies against the neuron-specific protein β-tubulin III, which is a marker of neurite development. The undifferentiated but stained cells showed low green fluorescence in the cytoplasm or neurite (Figure 2c), indicating that the marker was not present within the cells. Figure 2b,d show the morphological changes observed in the SH-SY5Y cell population throughout the differentiation period. Greenish fluorescence was observed in differentiated cells’ cytoplasm and neurites (Figure 2d), indicating a high level of β-tubulin III expression in both the cytoplasm and neurites of differentiated SH-SY5Y cells. Similar findings have been reported in previous studies [30,31], in which RA treatment resulted in neurite extension in SH-SY5Y cells. Phase contrast microscopy confirmed the success of the differentiation process. Consequently, differentiated cells were used throughout the experiment.

### 2.3. Cytotoxicity Effect of PMEE and H_2_O_2_ on SH-SY5Y Viability

**Figure 3:**
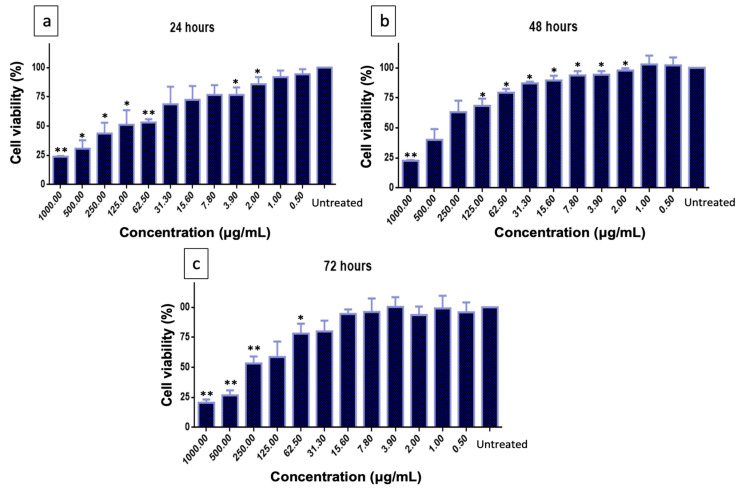
Cytotoxicity of PMEE on differentiated SH-SY5Y cells. (**a**) Viable cells after 24 h, (**b**) after 48 h, and (**c**) after 72 h. The values are the means of three independent trials (n = 3) and means with asterisks differed significantly (* *p* < 0.05, ** *p* < 0.01) with the untreated control.

**Figure 4:**
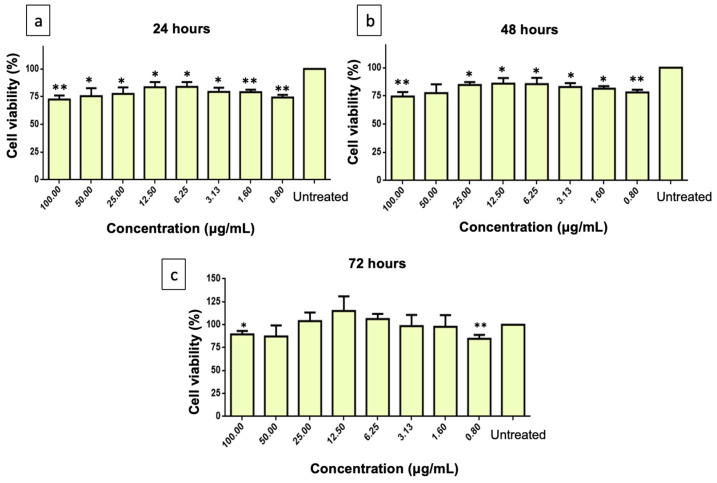
Cytotoxicity of curcumin on differentiated SH-SY5Y cells. (**a**) Cell viability after 24 h, (**b**) 48 h and (**c**) 72 h. The values are the means of three independent trials (n = 3) and means with asterisks differed significantly (* *p* < 0.05, ** *p* < 0.01) with the untreated control.

The MTT assay was used to determine cell viability in differentiated SH-SY5Y cells. The cells were exposed to PMEE at various concentrations (0.5 to 1000 μg/mL) for 24, 48, and 72 h. As shown in Figure 3, the PMEE-treated cells were viable across the concentrations used in a time-dependent manner. A similar pattern was observed in cells treated with curcumin at various concentrations (0.8 to 100 μg/mL) for 24, 48, and 72 h (Figure 4). As described in Section 3.6, the cytotoxicity of H_2_O_2_ was also determined by exposing differentiated cells to H_2_O_2_ at various concentrations (7.8 to 1000 μM/mL) for 4 h. In 4 h, 220 μM H_2_O_2_ caused approximately 50% cell death, according to the results obtained. Therefore, it was selected as the concentration of H_2_O_2_ to challenge PMEE pre-treated cells in the subsequent experiments. According to studies, exposing differentiated SH-SY5Y cells to cytotoxic agents such as hydrogen peroxide for a predetermined period results in oxidative stress and cell death. Several prior studies have demonstrated that H_2_O_2_ has a cytotoxic effect on differentiated SH-SY5Y cells used as a model for neuroprotection research [31,32]. Furthermore, it was observed that the use of *P. minus* extract at doses of up to 500 µg/mL did not exhibit any harmful effects on normal human lung fibroblast cell line (Hs888Lu) [33]. Ghazali et al. [34] studied the antiproliferative effect of various solvent extracts of *P. minus* using in vitro MTT assay against HepG2, WRL68, HeLA, HCT 116, MCF-7 and Chang cell lines. The ethanol extract showed lowest IC_50_ of 32.25 ± 3.72 μg/mL towards HepG2 cell lines with minimum toxicity in WRL68 normal embryonic liver cells whereas methanol extract showed moderate antiproliferative activity against HCT 116 cell lines (IC50 = 56.23 ± 3.2 μg/mL) [34]. *P. minus* had cytotoxic effects on cancer cells while demonstrating minimal toxicity towards normal cells. This shows that P. minus exhibits a selective effect in safeguarding normal cells.

### 2.4. Neuroprotective Effect of PMEE against H_2_O_2_-Induced Cytotoxicity

**Figure 5:**
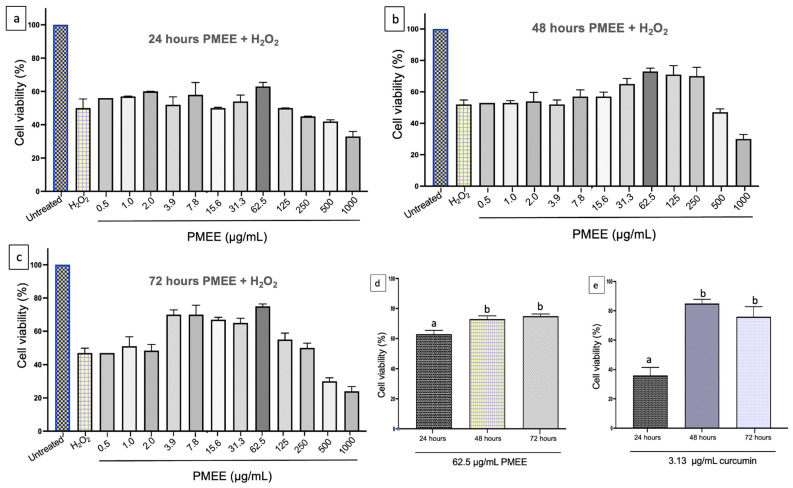
PMEE has a concentration-dependent neuroprotective effect. The differentiated SH-SY5Y cells were pre-treated with PMEE (0.5–1000 g/mL) for (**a**) 24, (**b**) 48, and (**c**) 72 h before being exposed to 220 μM H_2_O_2_ for 4 h. (**d**) A total of 62.5 μg/mL of PMEE and (**e**) 3.13 μg/mL of curcumin plus 4 h of exposure to 220 μM of H_2_O_2_. The values are the means of three independent trials (n = 3) and the means with different alphabets vary significantly (*p* < 0.05).

Damage to neurons resulting from oxidative stress (primarily reactive oxygen species) is one of the leading causes of neurodegenerative diseases [35]. To evaluate the neuroprotective effect of PMEE and curcumin against H_2_O_2_-induced cell death, the differentiated SH-SY5Y cells were pre-treated with a range of PMEE (0.5 to 1000 μg/mL) and curcumin (0.8 to 100 μg/mL) for 24, 48, and 72 h. Then, the pre-treated cells were exposed to IC_50_ of H_2_O_2_ (220 μM) for 4 h. MTT assay showed that H_2_O_2_ inhibited the cells’ viability, while pre-treatment of the cells with PMEE provided protection to the cells against the cytotoxic effect of H_2_O_2_ across the experimental period (Figure 5a–c) when compared to untreated control cells. However, pre-treatment with 62.5 μg/mL of PMEE demonstrated the highest viability against H_2_O_2_ especially after 48 and 72 h (Figure 5d). Moreover, 3.13 μg/mL of curcumin demonstrated the highest viability effect on the differentiated cells after 48 h of pre-treatment (Figure 5e). Hence, they were selected as working concentrations for the rest and standard control in the subsequent experiments.

The potential utilization of herbal medicines as a novel preventative neuroprotective strategy in the context of neurodegenerative illnesses is a subject of interest. These natural therapies could be explored for their applicability in individuals who are at risk of developing such conditions [36]. Exogenous antioxidants have been shown in studies to help prevent oxidative damage by reducing ROS production in cells, increasing their chances of survival [3,5]. The current findings demonstrated how different concentrations of PMEE increased the viability of differentiated neurons in a time and dose-dependent manner. However, 62.5 μg/mL of PMEE demonstrated the greatest potential in that regard, indicating a high capability for reducing susceptibility caused by hermetic response in the cells. The effect was clearly greater after 48 and 72 h of treatment than after 24 h. This is due to the long-term effect on endogenous defensive mechanisms, which reduces the cells’ vulnerability to attack from endogenous cytotoxic agents. Previous research found that an ethyl acetate extract of *P. minus* has a selective antiproliferative effect on HepG2 cells while having little cytotoxicity on normal liver cells [34].

### 2.5. PMEE Pre-Treatment Influenced Gene Expressions in Nrf2/ARE Pathway

**Figure 6:**
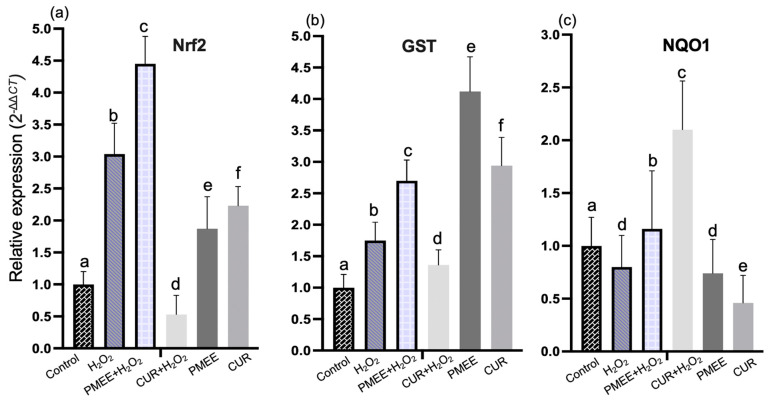

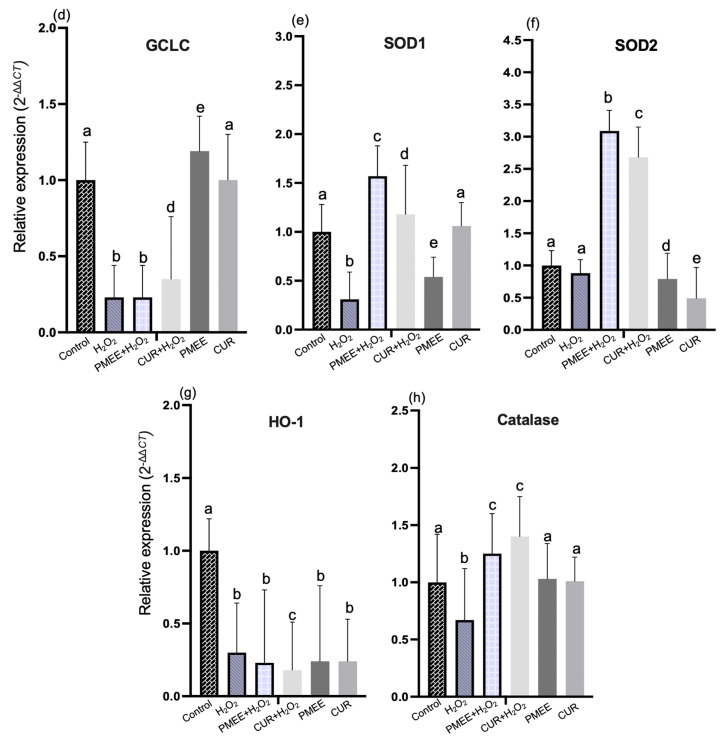
The mRNA expression of (**a**) Nrf2, (**b**) GST, (**c**) NQO1, (**d**) GCLC, (**e**) SOD1, (**f**) SOD2, (**g**) HO-1 and (**h**) catalase in the Nrf2/ARE signaling pathway. Control: untreated cells; H_2_O_2_: cells induced with 300 μM hydrogen peroxide for 4 h; PMEE + H_2_O_2_: cells pre-treated with PMEE for 48 h + 4 h exposure to H_2_O_2_; CUR+H_2_O_2_: cells pre-treated with curcumin for 48 h + 4 h exposure to H_2_O_2_; PMEE: cells treated with only PMEE; CUR: cells treated with only curcumin. The value represents fold changes between control (untreated cells) and treatment groups. Data were expressed as the mean ± SD of triplicate experiments; values with different letters alphabets are significantly different from one another and vice versa (*p* < 0.05).

The expression level of Nrf2, NQO1, SOD1, SOD2, and catalase under the Nrf2/ARE signaling pathway increased significantly (*p* < 0.05) in cells exposed to PMEE plus 4 h of H_2_O_2_ compared to cells treated with PMEE alone (Figure 6). The pre-treatment of differentiated neuron cells with PMEE increased the expression of these genes above the normal level. When Nrf2 is released from the cytoplasm, it translocates to the nucleus as a transcription factor. The factor binds to the antioxidant response element (ARE) to form a complex that binds to the promoter region of phase II antioxidant genes to initiate transcription of the phase II antioxidant proteins, which play a role in counteracting the toxic effects of free radicals and protecting neurons from damage and death [37]. Though under oxidative stress, the presence of PMEE enhanced the expression of Nrf2 genes and their translocation to the nucleus. In addition, the increased expression of NQO1 in cells pre-treated with PMEE suggests a potential gene-level neuroprotective effect of PMEE.

The mRNA level of the GST gene increases significantly (*p* < 0.05) when the cells are pre-treated with PMEE with or without an H_2_O_2_ challenge compared to the H_2_O_2_ control group (Figure 6b). GST proteins are significant antioxidant enzymes that regulate stress-induced signaling pathways and are essential for scavenging the free radicals produced by cells [38]. Increased expression or activity of this enzyme indicated improved antioxidant activity. PMEE’s ability to prevent neuronal death caused by oxidative stress was indicated by its ability to increase GST expression. PMEE pre-treatment prior to H_2_O_2_ exposure resulted in a significant (*p* < 0.05) decrease in the mRNA expression level of the GCLC gene in differentiated SH-SY5Y cells compared to PMEE alone (Figure 6d). The GCLC encodes glutamate-cysteine ligase, a phase II antioxidant marker with extraordinary antioxidant activity in humans and other closely related species [31]. In reaction to oxidative stress as well as other inflammatory factors, the level of GCLC increases to neutralize the harmful environmental effect and ensure the survival of affected cells. However, inducing H_2_O_2_ for 4 h in PMEE pre-treatment differentiated SH-SY5Y cells did not enhance the expression level of GCLC.

SOD1 and SOD2 gene expression increased significantly (*p* < 0.05) in differentiated cells treated with PMEE prior to H_2_O_2_ exposure, as compared to control cells and cells treated with PMEE alone (Figure 6e,f). The two genes code for the enzyme’s superoxide dismutase 1 and 2, which are abundant in neural tissue. Part of the physiological and antioxidant significance of these proteins is their ability to regulate superoxide concentration by converting superoxide to hydrogen peroxide, a substrate for another phase II enzyme that is less damaging to the former [39]. This phenomenon facilitates neuron cell survival in a toxic environment. In contrast, the expression of the HO-1 gene decreased significantly (*p* < 0.05) in all treatment groups (Figure 6g) compared to untreated cells. This indicates that treatment exposure had no effect on the expression of HO-1 in differentiated SH-SY5Y cells. Moreover, after 48 h, the level of mRNA for catalase gene expression increased significantly (*p* < 0.05) in PMEE pre-treated cells plus 4 h of H_2_O_2_ exposure compared to its expression in H_2_O_2_ control and PMEE-treated cells (Figure 6h). Catalase plays a crucial antioxidant role in the body by converting harmful substances, such as cellular hydrogen peroxide, into less toxic forms (oxygen or water) [40].

### 2.6. PMEE Pre-Treatment Influenced Gene Expressions in NF-κB/IκB Pathway

**Figure 7:**
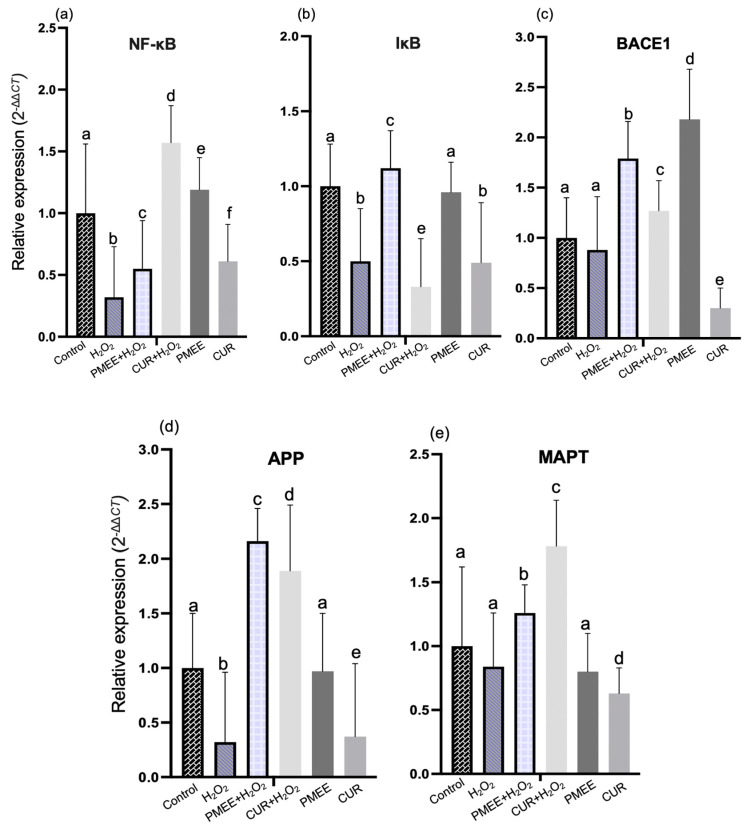
The mRNA expression of (**a**) NF-κB, (**b**) IκB, (**c**) BACE1, (**d**) APP and (**e**) MAPT in NF-κB/IκB signaling pathway. Control: untreated cells; H_2_O_2_: cells induced with 300 μM hydrogen peroxide for 4 h; PMEE+H_2_O_2_: cells pre-treated with PMEE for 48 h + 4 h exposure to H_2_O_2_; CUR+H_2_O_2_: cells pre-treated with curcumin for 48 h + 4 h exposure to H_2_O_2_; PMEE: cells treated with only PMEE; CUR: cells treated with only curcumin. The value represents fold changes between control (untreated cells) and treatment groups. Data were expressed as the mean ± SD of triplicate experiments; means with different letters denote significant differences with one another and vice versa (*p* < 0.05).

In oxidative stress conditions with or without PMEE and curcumin, the mRNA expression level of genes involved in the NF-κB/IκB -mediated neuropathological pathway is drastically altered. This pathway is influenced by NF-κB, IκB, BACE1, APP, and MAPT genes. All gene expression levels were significantly (*p* < 0.05) higher in PMEE-treated cells than in H_2_O_2_ control cells (Figure 7). Pre-treatment of differentiated SH-SY5Y cells with PMEE for 72 h inhibited the overexpression of the NF-κB gene significantly (*p* < 0.05) when the cells were exposed to an H_2_O_2_-induced toxic environment for 4 h, as compared to its high expression in the PMEE-only treatment group (Figure 7a). Reportedly, inhibition of NF-κB mediates neuroprotection in neurodegenerative diseases such as Alzheimer’s and multiple sclerosis [41]. The present findings suggested that PMEE could selectively inhibit the expression of NF-κB subunits, which are accountable for the transcription of inflammatory markers that induce neuronal damage and death. PMEE pre-treatment prior to H_2_O_2_ exposure significantly (*p* < 0.05) increased the level of mRNA for the IB gene in differentiated neuron cells compared to H_2_O_2_ control cells (Figure 7b). The IκB gene encodes the IκBα protein, which prevents the production of inflammatory cytokines. IκBα and NF-κB interact in two ways: by retaining the transcription factor (NF-κB) in the cytoplasm and by inhibiting its DNA binding in the nucleus. PMEE perhaps strengthened the interaction between the two molecules by preventing phosphorylation of IκBα which leads to the release of NF-κB and its translocation to the nucleus.

The expression level of the BACE1 gene decreased significantly (*p* < 0.05) in PMEE-treated cells prior to 4 h of H_2_O_2_ exposure when compared to the expression in H_2_O_2_ control cells (Figure 7c). In Alzheimer’s and Down syndrome disease models, inhibiting the activity of or silencing this gene was associated with neuroprotection [42]. Thus, the genotoxic effect of PMEE on the expression of the BACE1 gene demonstrated the compound’s potential to inhibit the functional BACE1 protein’s activity. In contrast, 48 h of PMEE pre-treatment increased APP and MAPT gene expression significantly (*p* < 0.05) in H_2_O_2_-treated control cells without PMEE pre-treatment (Figure 7d,e).

### 2.7. PMEE Pre-Treatment Influenced Gene Expressions in the MAPK Pathway

**Figure 8:**
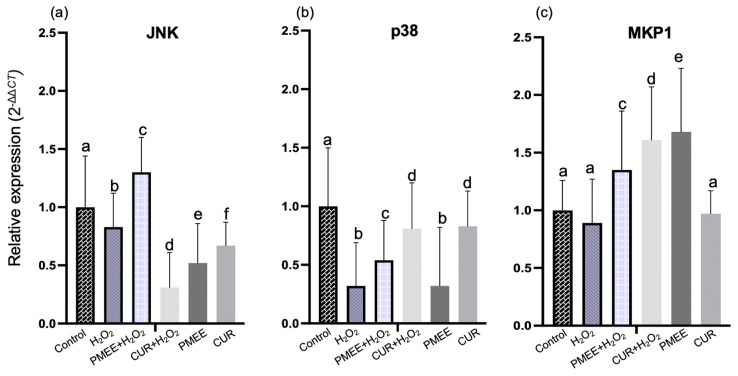

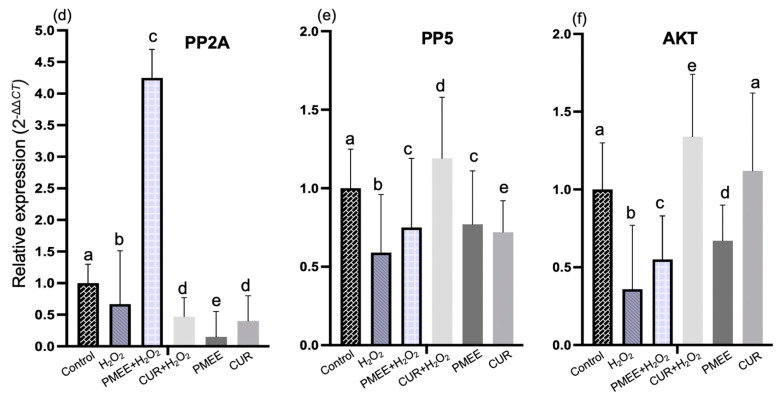
The mRNA expression of (**a**) JNK, (**b**) p38, (**c**) MKP1, (**d**) PP2A, (**e**) PP5 and (**f**) AKT in MAPK signaling pathway. Control: untreated cells; H_2_O_2_: cells induced with 300 μM hydrogen peroxide for 4 h; PMEE+H_2_O_2_: cells pre-treated with PMEE for 48 h + 4 h exposure to H_2_O_2_; CUR+H_2_O_2_: cells pre-treated with curcumin for 48 h + 4 h exposure to H_2_O_2_; PMEE: cells treated with only PMEE; CUR: cells treated with only curcumin. The value represents fold changes between control (untreated cells) and treatment groups. Data were expressed as the mean ± SD of triplicate experiments; means with different letters denote significant difference (*p* < 0.05).

PMEE pre-treatment prior to H_2_O_2_ exposure led to a significant (*p* < 0.05) increase in the expression of JNK, p38, and PP2A genes in differentiated cells compared to the expression in H_2_O_2_ control cells and PMEE only group (Figure 8a,b,d). Curcumin (positive control) could not prevent the genotoxic effect of H_2_O_2_ on the expression level of JNK, unlike PMEE-treated cells. JNK is a gene that codes for c-Jun N-terminal kinases-3 (JNK3), a neuron-specific isoform protein that plays an essential role in the pathophysiology of a variety of neurological disorders. The upregulation of JNK is activated by H_2_O_2_-induced cellular stress. In addition, the p38 gene is a member of the mitogen-activated protein kinase (MAPK) family that plays a crucial role in MAPK pathway-mediated apoptosis [43]. Previous research has demonstrated the neuroprotective effects of PP2A against neuronal apoptosis in cases of traumatic brain injury, acute ischemia, and neurodegenerative disease [44,45,46,47]. After 4 h of exposure to H_2_O_2_, this indicated that PMEE protects neuroblastoma SH-SY5Y cells from oxidative stress by increasing JNK, p38, and PP2A gene expressions. Meanwhile, the expression level of MKP1 and AKT decreased significantly (*p* < 0.05) in the presence of PMEE pre-treatment prior to H_2_O_2_ exposure compared to PMEE only treatment group (Figure 8c,f). In contrast, the presence of PMEE did not increase the expression of PP5 under oxidative stress conditions in cells treated with PMEE alone (Figure 8e).

### 2.8. PMEE Pre-Treatment Increased the Expression of Acetylcholine (ACH) in SH-SY5Y Differentiated Cells

**Figure 9:**
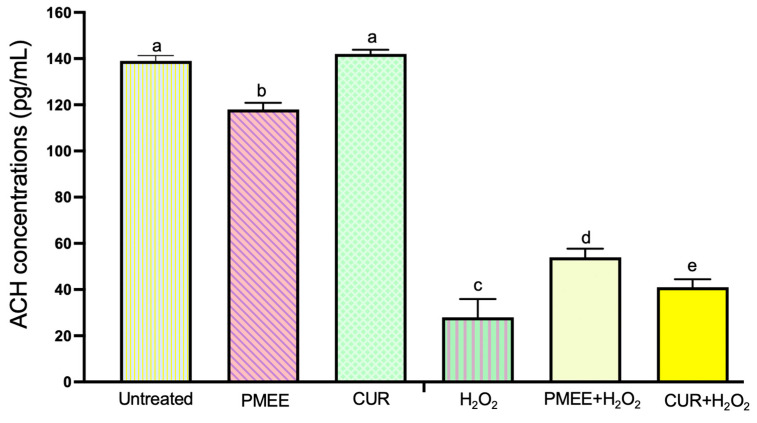
Acetylcholine (ACH) level in the supernatant of differentiated SH-SY5Y cells. The ACH level of cells induced with H_2_O_2_ increased in both PMEE and CUR-treated cells. Control: untreated cells; H_2_O_2_: cells induced with 300 μM hydrogen peroxide for 4 h; PMEE+H_2_O_2_: cells pre-treated with PMEE for 48 h + 4 h exposure to H_2_O_2_; CUR+H_2_O_2_: cells pre-treated with curcumin for 48 h + 4 h exposure to H_2_O_2_; PMEE: cells treated with only PMEE; CUR: cells treated with only curcumin. The value represents fold changes between control (untreated cells) and treatment groups. Data were expressed as the mean ± SD of triplicate experiments; means with different letters denote significant differences (*p* < 0.05).

It has been demonstrated that ACH inhibits the production of ROS during oxidative stress. Therefore, we examined the effects of ACH concentration before or after H_2_O_2_ treatment on the differentiated SH-SY5Y cells using ELISA. The ACH level in the differentiated SH-SY5Y cells was significantly (*p* < 0.05) increased in PMEE or curcumin pre-treatment prior to 4 h of H_2_O_2_ exposure compared to that in H_2_O_2_ control cells as shown in Figure 9. In the present study, PMEE and curcumin have been shown to possess positive effect by increasing the expression of ACH under oxidative conditions.

The potential utilization of herbal medicines as a novel preventative neuroprotective strategy in the context of neurodegenerative illnesses is a subject of interest. These natural therapies could be explored for their applicability in individuals who are at risk of developing such conditions [36]. The AChE is an enzyme that is responsible for the metabolism of the neurotransmitter ACH, and inhibiting AChE can have therapeutic (e.g., Alzheimer’s disease drugs) or neurotoxic effects (e.g., pesticides). Patients with coronary artery disease and Alzheimer’s disease pathogenesis had elevated ACH gene expression. The finding reported by Işık and Beydemir [48] suggested that an increase in cellular AChE release results in the formation of neurotoxic β-amyloid plaques and may cause neurodegenerative diseases. According to the cholinergic hypothesis, the inhibition of AChE, an enzyme that catalyzes acetylcholine hydrolysis, increases the levels of ACH in the brain, thus improving cholinergic functions in Alzheimer’s disease patients. The use of AChE inhibitors has proven to be an effective approach in the management of neurological conditions such as Alzheimer’s disease [49]. Therefore, one of the important strategies for treating neurological disease is to maintain the levels of ACH through the inhibition of AChE [50].

The inhibitory actions of the aqueous and methanolic extracts of *P. minus* leaves were observed on the AChE enzyme [15,51]. A prior research conducted by George et al. [15] demonstrated that the aqueous extract of *P. minus* had inhibitory effects on cholinesterase activity, with an IC_50_ value of 0.04 mg/mL and a maximal inhibition rate of 68%. The findings of this study indicate that *P. minus* exhibits antioxidant and anticholinesterase properties, and it has been observed to improve cognitive function in vivo and indicates that the extract possesses neuroprotective effects. The AChE inhibitory activity of *P. istanbulicum*, was observed in a dose-dependent manner. The ethanolic extract of *P. istanbulicum* demonstrated the highest level of inhibition against AChE, with an inhibition rate of 88.2 ± 3.44% as compared to other species of polygonum such as *P. patulum* subsp. *Pulchellum*, *P. aviculare* and *P. lapathifolium* [52]. In this present study, *P. minus* showed promising potential as a therapeutic intervention for Alzheimer’s disease due to the observed favorable impact of PMEE on the upregulation of ACH concentrations under oxidative conditions.

### 2.9. Molecular Docking

The results of molecular docking between PMEE’s identified compounds and protein AChE (Protein Data Bank ID: 4EY6) are shown in Table 2. The complexes exhibited comparable binding interaction energy values in the range of −9.5 to −5.8 kcal/mol. The complexes of AChE and quercitrin, aloe-emodin, afzelin and citreorosein showed more negative binding values compared to other compounds, i.e., quercitrin showed the highest docking score at −9.5 kcal/mol followed by aloe-emodin, afzelin, and citreorosein at −9.4, −9.3 and −9.0 kcal/mol, respectively. Lower binding affinity depicts better ligand receptor interaction as well as higher docking score against AChE.

**Figure 10:**
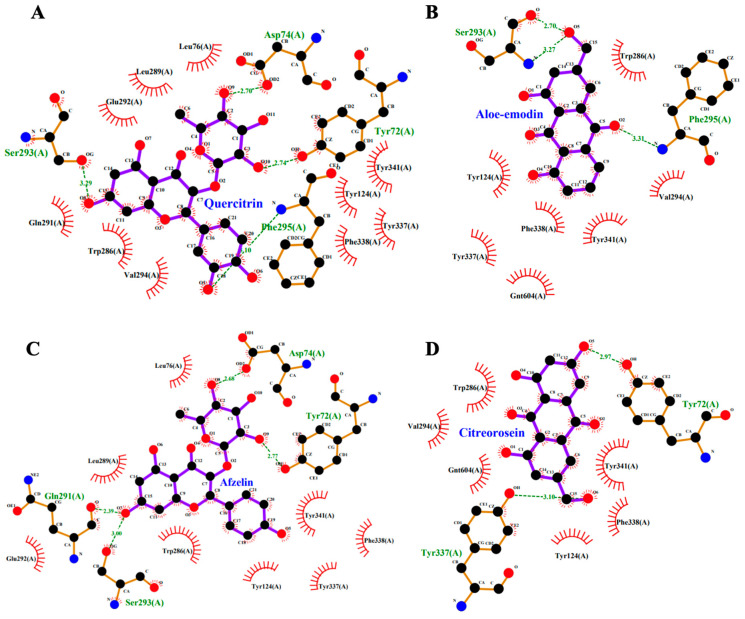
Two-dimensional (2D) interactions of AChE and selected PMEE’s identified compounds. (**A**) Interactions of quercitrin and AChE. (**B**) Interactions of aloe-emodin and AChE. (**C**) Interactions of afzelin and AChE. (**D**) Interactions of citreorosein and AChE.

**Figure 11:**
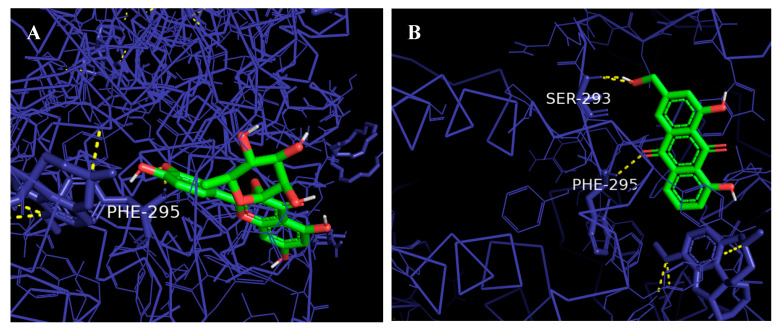

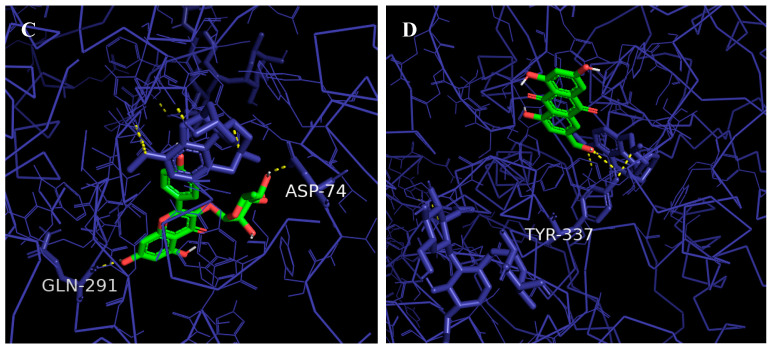
Three-dimensional (3D) interactions of AChE and selected PMEE’s identified compounds. Ligands are illustrated in green, AChE protein in dark blue, and hydrogen bonds are depicted in yellow dots. (**A**) Interactions of quercitrin and AChE. (**B**) Interactions of aloe-emodin and AChE. (**C**) Interactions of afzelin and AChE. (**D**) Interactions of citreorosein and AChE.

Figure 10 and Figure 11 demonstrate results from ligand–protein interaction between AChE and four most active compounds indicated by binding affinity scores (kcal/mol). It showed that the interacting amino acids of AChE and quercitrin were found to be Tyr72, Asp74, Ser 293, and Phe295. Meanwhile, ligand–protein interaction between AChE and the following compounds showed that the interacting amino acids at the active site were found to be Ser293 and Phe295 (aloe-emodin), Tyr72, Asp74, Ser 293, and Gln291 (afzelin), and Tyr72 and Tyr337 (citreorosein). Ligplot analysis showed 2D structure where the hydrogen bonding is in green dashed lines and hydrophobic interaction is in red arcs between AChE with all the different ligands. Furthermore, Pymol analysis showed 3D structure of ligand–protein interactions where the green color indicates the ligand and red color indicates interacting amino acids of the protein.

## 3. Materials and Methods

### 3.1. Plant Collection and Preparation of Ethanolic Extract

*P. minus* leaves (5 kg) were collected from an experimental plot of INBIOSIS. Original samples (10 kg) were collected from Cameron Highland, Malaysia and a voucher specimen was deposited in the UKMB Herbarium, Universiti Kebangsaan Malaysia. Specimens were identified by a taxonomist and further confirmed by ITS sequencing. *P. minus* leaves (1 kg) were air dried at room temperature (+27 °C) and powdered using a blender (230–250 mesh). Approximately 360 g of leaf powder were soaked in 7.2 L ethanol. The extraction was performed with ratio 1:20 (*w*/*w*) for 72 h at room temperature. The mixture was filtered, and the filtrate was concentrated using an EYELA OSB-2100 rotary vacuum evaporator model N-11005-WD until complete dryness at 40 °C. Subsequently, the semi-dried ethanol extract was freeze dried using Labconco freeze dryer model 74200-30. The extract was referred to as *P. minus* ethanolic extract (PMEE) and was utilized in subsequent analysis.

### 3.2. Liquid Chromatography–Mass Spectrometry (LC–MS/MS)

LC–MS/MS was performed with slight modifications to the method described by Bingol and Bursal [53]. The separation was performed using Thermo Scientific (C18 column (AcclaimTM RepMap RSLC, 75 µm × 15 µm, 2 µm, 100 A) on an Dionex UltiMate 3000 UHPLC system (Thermo Scientific, Waltham, MA, USA). The dry ethanolic extract was dissolved in HPLC-grade methanol. As an internal benchmark, umbelliferon was used. The sample injection volume was 20 μL, and the temperature and flow rate of the column were 60 °C and 0.3 mL/min. The mobile phases were 0.1% formic acid dissolved in water (mobile phase A) and acetonitrile (mobile phase B). The elution was carried out with a 35 min gradient beginning with an increase from 0 to 5% B in the first two minutes, then to 40% B in the next two minutes, and finally to 95% B in the following 16 min. At 95% B, the mixture was held for 2 min prior to an increase of 0.1 min to 100% B. At 100 percent B, the mixture was held for four minutes before dropping to 5 percent B in two minutes. The column (C18, Thermo Scientific) was then reconditioned with the initial gradient for seven minutes. MS/MS analysis was performed using a MicroTOF-QIII (Bruker, Bremen, Germany) system equipped with an electrospray ionization (ESI) source operating in a positive mode of ionization. The nitrogen drying gas was set to 45 psi with a flow rate of 8 L min1 and a temperature of 200 °C. The voltage of ESI spray was fixed at 4.5 kV, and the voltage of the fragmentor was set at 200 V. For the mass range of 50–1500 *m*/*z*, ionization-mode mass spectrum data were recorded. MS-DIAL version 3.70 was utilized for all compound identifications.

### 3.3. Cell Lines and Cell Cultures

The human neuroblastoma SH-SY5Y cell line was purchased from ATCC, Manassas, VA, USA, (ATCC^®^ CRL-2266^TM^). In this experiment, passage 5 cell lines were used. Dulbecco’s Modified Eagle and Hams’ 13 media were combined to maintain the cells (DMEM/Hams’ F12) (Nacalai, Kyoto, Japan), with 10% fetal bovine serum (FBS), 1% penicillin and streptomycin) and incubated at 37 °C in 5% CO_2_ with 95% humidified atmospheric air.

### 3.4. Neuronal Differentiation of SH-SY5Y Cells

Differentiation of SH-SY5Y cells was achieved in accordance with the stipulated protocol outlined in Jaafaru et al. [31]. According to the predetermined protocol specified by Jaafaru et al. [31] SH-SY5Y cells were successfully differentiated into neuron-like cells. In brief, the cells were seeded in a 6-well plate at a density of 1 × 10^5^ cells/well. Following a 24 h incubation period, each well was added 2 mL of DMEM/F12 media containing 3% heat-inactivated FBS and 10 µM retinoic acid (RA). This was performed in the dark with the incubator set to 37 °C with 5% CO_2_. For a period of seven days, the differentiation media was changed every two days. RA-induced differentiation was examined under phase contrast using an inverted light fluorescence microscope (Zeiss Axio Vert A1, Göttingen, Germany) fitted with an image acquisition system (AxioCam MRm, Göttingen, Germany), and multiple images were taken independently.

### 3.5. Immunocytochemistry (ICC) Assay

To further ascertain the differentiation of SH-SY5Y cells into full neuronal cells by retinoic acid (RA), ICC was conducted according to the protocol described by Jaafaru et al. [31]. The cells were differentiated as previously mentioned after being seeded in 24-well plates at a density of 2 × 10^4^ cells/well. The differentiated cells were washed three times with cold phosphate buffer saline pH 7.4, at 25 °C followed by incubation with 300 µL fixation solution (4% Paraformaldehyde, 1M NaOH and PBS) at 25 °C for 30 min and washed with PBS thereafter. Permeation solution (1% Triton X-100 and 99% PBS) and blocking (0.3% bovine serum albumin, 10% goat serum, 10% tween 20 and PBS) solution were incubated with the cells at 25 °C for 15 min and 30 min, accompanied with washing at each stage. Antibody for class III β-tubulin (Tuj-1), a cytoplasmic neuron-specific protein, was added in ratio of 1:200 blocking solution with subsequent overnight incubation at 4 °C. The cells were washed with PBS the following day and incubated with Alexa fluoropore-488 secondary antibody conjugate (1:200) in the dark at 25 °C for 2 h. Then, the cells were incubated with nuclear counterstaining dye (DAPI dye) for 10 min before images were taken using an inverted light fluorescence microscope (Zeiss Axio Vert A1, Germany) with an image acquisition system (AxioCam MRm, Göttingen, Germany).

### 3.6. Cytotoxicity of PMEE on the SH-SY5Y Cells

The effect of PMEE on cell viability on differentiated SH-SY5Y cells were assessed using the MTT reduction assay, as modified by Jaafaru et al. [31]. In a 96-well plate, 1 × 10^4^ SH-SY5Y cells were seeded, and they underwent a seven-day period of differentiation process as outlined in Section 3.4. The cells were treated with serially diluted concentrations of PMEE (0.5–1000 µg/mL) for 24, 48, and 72 h to determine how PMEE affected cell viability. The plate was incubated in the dark for four hours after 20 µL addition of MTT solution and then 200 µL of DMSO was added after removal of cell medium to dissolve the formazan that had formed in the wells. Absorbance was measured immediately at 540 nm using a microplate reader. Similar analysis was conducted for H_2_O_2_ cytotoxic effect, in which 1000 µM concentration was serial diluted to 7.8 µM and the optical density was used to evaluate the IC_50_ of H_2_O_2_ used in the present study.

### 3.7. Neuroprotection of PMEE on the SH-SY5Y Cells

Differentiated SH-SY5Y cells were pre-treated with serial dilutions of PMEE to determine the neuroprotective activity of the PMEE in time-dependent manner prior to 4 h challenged by 220 µM (IC_50_) H_2_O_2_, followed by addition of 20 µL and 200 µL of MTT and DMSO reagent, respectively. Curcumin was used as positive control. The absorbance reading was measured immediately at 540 nm using a microplate reader.

### 3.8. PMEE Pre-Treatment and H_2_O_2_ Exposure

The differentiated neuronal cells were seeded in T25 flasks at a density of 1 × 10^3^ cells/mL and underwent differentiation as outlined in Section 3.4. For 48 h, the cells were pre-treated separately with PMEE (6.25 µg/mL) or curcumin (3.13 µg/mL). Prior to bioassay analyses, the pre-treated cells were exposed to 220 µM H_2_O_2_ for 4 h.

### 3.9. Gene Expression Study of PMEE-Treated SH-SY5Y Cells

After differentiation and treatment, genomic RNA was extracted using an RNA extraction kit (NucleoSpin RNA Plus, Macherey, Düren, Germany) in accordance with the manufacturer’s instructions. The concentration and purity of the isolated RNA were evaluated using Nanodrop spectrophotometer (Thermo Scientific Nanodrop, NanoDrop Technologies, Wilmington, DE, USA). The cDNA was synthesized from one μg of RNA using the HiScript III First Strand cDNA Synthesis kit +gDNA wiper (R312-02, Vazyme, Nanjing, China). Meanwhile, the qPCR was conducted using Maxima SYBR green qPCR Master Mix (Q712-02, Vazyme) according to the manufacturer’s instructions. The nucleotide primer sequences used in this study were presented in Table 3. The primers were synthesized by Bio3 Scientific Sdn. Bhd. (Puchong, Malaysia). The glycerldehyde-3-phosphate dehydrogenase (GAPDH), a housekeeping gene, was used as an internal reference (forward 5′-GTCATCCCTGAGCTGAACGG-3′, reverse 5′-AAGTGGTCGTTGAGGGCAAT-3′). Each gene was amplified three times using RT-qPCR. The amplification parameters were as follows: 95 °C for 30 s, 95 °C for 5 s, and 60 °C for 31 s for a total of 40 cycles. Using the 2-∆∆Cq method, the quantification values were subsequently calculated and analyzed. Ratio in untreated cells (negative control) was assigned as 1.

### 3.10. Acetylcholine (ACH) Enzyme-Linked Immunosorbent Assay (ELISA)

To detect the ACH release in the culture medium, an ACH enzyme-linked immunosorbent assay (ELISA) (E-EL-0081, Elabscience, Houston, TX, USA) was performed according to the manufacturer’s instructions. The cells were cultured in 25 cm^2^ flask at a density of 1 × 10^6^ cells/flask and were differentiated as described in Section 3.4. After 7 days, each flask’s cell medium was collected and centrifuged for 20 min at 1000× *g* and 4 °C. The cell supernatant was collected for the assay. The absorbance value was determined at 550 nm, with the color intensity proportional to the ACH concentration. The concentration of ACH in samples was determined by comparing the absorbance of the samples to the standard curve.

### 3.11. Molecular Docking

Molecular docking study was conducted to test the binding affinity of PMEE’s identified compounds to AChE enzyme residues. AChE (PDB ID: 4EY6) was retrieved as a PDB file from the RCSB Protein Data Bank (http:/www.rcsb.org/pdb/, accessed on 15 August 2023). The Auto Dock Tools (version 1.5.7) was used to prepare protein. Crystallographic waters were removed, polar hydrogens were added to a macromolecule, along with Kollman charges. To get the best conformational docking state, a grid box covering the active site residues of the target protein was created. Using AutoDock Tools 1.5.7, the docking search site was established where ligands could investigate potential binding interactions with AChE [54]. The 3D cuboidal AutoGrid box’s center was set to (x: 12.3199, y: 42.071, z: 28.832), and its dimensions were set to (x: 24 y: 20 z: 20) for the number of points. The molecular docking runs were carried out using command prompt. The AutoDock Vina software, target receptor and ligand pdbqt files, configuration text file, and intended destination of output data were all supplied in the docking command line. The resulting AutoDock Vina output files in pdbqt format contained the generated poses as well as text data listing the relevant poses’ binding energies [55]. The binding affinity measured in terms of binding energy (kcal/mol) and the visualization of binding conformation for each docking mode using PyMOL were the two results from molecular docking.

### 3.12. Statistical Analysis

Data are presented as the mean standard deviation, and differences between means of each group were determined by one-way analysis of variance (ANOVA) with Tukey’s multiple comparison, using Graph Pad Prism 9 (GraphPad Software, Inc., San Diego, CA, USA). The 95% confidence interval was considered, thus *p* < 0.05 signified statistical significance.

## 4. Conclusions

This study revealed the ability of PMEE to halt ROS generation due to oxidative stress induced by H_2_O_2_. The findings showed that the demonstrated effects were coordinated through the Nrf2/ARE, NF-κB/IκB, and MAPK signaling pathways, thus concluding that PMEE confers neuroprotection against oxidative stress in differentiated SH-SY5Y cells. Quercitrin had the best docking score compared to the other compounds found in PMEE, which had lower docking scores. The present study suggests that PMEE may be a potential therapeutic agent for the treatment of neurodegenerative disorders associated with oxidative stress. The results of our study provide a justification for further investigation into the application of PMEE in animal models of neurodegenerative disorders, in order to assess their safety and effectiveness. Additionally, this would serve as a fundamental basis for subsequent clinical investigations.

## Figures and Tables

**Table 1:** List of compounds identified in PMEE by LC–MS/MS analysis.

No	Identified Compounds	Retention Time (min)	Molecular Formula	Molecular Weight (g/mol)
1	Quinic acid	2.540	C_7_H_12_O_6_	191.06
2	Gallic acid	6.456	C_7_H_6_O_5_	169.01
3	2,3-Dihydroxybenzoic acid	8.170	C_7_H_6_O_4_	153.02
4	(−)-epicatechin	8.686	C_15_H_14_O_6_	291.09
5	5-Hydroxy-2-penten-1-yl-3-oxocyclopentyl acetic acid	8.753	C_12_H_18_O_4_	227.13
6	Caffeic acids	9.142	C_9_H_8_O_4_	179.04
7	Loliolide	9.506	C_11_H_16_O_3_	197.12
8	Quercitrin	9.673	C_21_H_20_O_11_	449.11
9	Naphtho[2,3-b]furan-9(4*H*)-1,4,8-bis(acetyloxy)-4a,5,6,7,8,8a-hexahydro-3,4a,5-trimethyl-, (4S,4aR,5S,8S,8aS)	9.823	C_19_H_24_O_6_	331.15
10	Carboxymethyl-cyclohexanecarboxylic acid	9.961	C_9_H_14_O_4_	185.08
11	Afzelin	10.073	C_21_H_20_O_10_	433.11
12	6-Hydroxy-3-isopropylidene-4a,5-dimethyl-4,4a,5,6,7,8-hexahydro-2(3*H*)-naphthalenone	10.157	C_15_H_22_O_2_	235.17
13	Feruloyltyramine	10.157	C_18_H_19_NO_4_	314.14
14	Daphnetin	10.362	C_9_H_6_O_4_	177.02
15	Esculetin	10.423	C9H_6_O_4_	179.03
16	Citreorosein	10.910	C_15_H_10_O_6_	287.05
17	Quercetin	11.428	C_15_H_10_O_7_	303.05
18	Aloe-emodin	12.250	C_15_H_10_O_5_	271.06
19	Kaempferol	12.250	C_15_H_10_O_6_	287.05
20	Rhamnetin	12.250	C_16_H_12_O_7_	317.06
21	(2*Z*)-4,6-dihydroxy-2-[(4-hydroxy-3,5-dimethoxyphenyl)methylidene]-1-benzofuran-3-1	12.516	C_17_H_14_O_7_	331.08
22	Eupatilin	12.516	C_18_H_16_O_7_	345.09
23	Corynoxeine	12.971	C_22_H_26_N_2_O_4_	383.20
24	Valerenic acid	13.062	C_15_H_22_O_2_	233.16
25	alpha-Cyperone	13.154	C_15_H_22_O	219.17
26	Dibutylphthalate	13.304	C_16_H_22_O_4_	279.16
27	(2*Z*)-2-[(*E*)-6-(hydroxymethyl)-2,4,8,10-tetramethyldodec-2-enylidene]-4-methylpentanedioic acid	13.847	C_23_H_40_O_5_	419.27
28	Prespatane	14.260	C_15_H_24_	205.19

**Table 2:** Binding affinity of PMEE’s identified compounds with acetylcholinesterase (AChE).

No.	PMEE’s Identified Compounds	Binding Affinity to AChE (kcal/mol)
1.	Quercitrin	−9.5
2.	Aloe-emodin	−9.4
3.	Afzelin	−9.3
4.	Citreorosein	−9.0
5.	alpha-Cyperone	−8.7
6.	Quercetin	−8.7
7.	Kaempferol	−8.6
8.	Rhamnetin	−8.6
9.	(2*Z*)-4,6-dihydroxy-2-[(4-hydroxy-3,5-dimethoxyphenyl) methylidene]-1-benzofuran-3-1	−8.5
10.	6-Hydroxy-3-isopropylidene-4a,5-dimethyl-4,4a,5,6,7,8- hexahydro-2(3*H*)-naphthalenone	−8.3
11.	Eupatilin	−8.3
12.	Feruloyltyramine	−8.0
13.	Naphtho[2,3-b]furan-9(4*H*)-1,4,8-bis(acetyloxy)-4a,5,6,7,8,8a- hexahydro-3,4a,5-trimethyl-, (4S,4aR,5S,8S,8aS)	−7.8
14.	(−)-epicatechin	−7.7
15.	Esculetin	−7.5
16.	Prespatane	−7.5
17.	(2*Z*)-2-[(*E*)-6-(hydroxymethyl)-2,4,8,10- tetramethyldodec-2-enylidene]-4-methylpentanedioic acid	−7.3
18.	Daphnetin	−7.3
19.	Valerenic acid	−7.3
20.	Caffeic acids	−7.0
21.	Corynoxeine	−7.0
22.	Dibutylphthalate	−7.0
23.	Loliolide	−6.6
24.	5-Hydroxy-2-penten-1-yl-3-oxocyclopentyl acetic acid	−6.5
25.	Carboxymethyl-cyclohexanecarboxylic acid	−6.5
26.	Gallic acid	−6.5
27.	2,3-Dihydroxybenzoic acid	−6.2
28.	Quinic acid	−5.8

**Table 3:** Gene name, accession number, forward and reverse primer sequences used in the real-time PCR analysis.

Gene and Accession No	Forward Primer	Reverse Primer
AKT [NM_005465.4]	AGGTGACACTATAGAATA AGACATTAAATTTCCTCGAA	GTACGACTCACTATAGGG AATCCTCATCATATTTTTCAGGT
APP [NM_000484.3]	AGGTGACACTATAGAATA CTGTGGCAGACTGAACATGC	GTACGACTCACTATAGGG ATCACCAACTAAGCAGCGGTA
BACE1 [NM_012104.4]	AGGTGACACTATAGAATA CGAGCTGGATTATGGT	GTACGACTCACTATAGGG AGGAGAGGGAGCTTGG
Catalase [NM_001752.3]	AGGTGACACTATAGAATA AGAAATCCTCAGACACATCT	GTACGACTCACTATAGGG AATGTCATGACCTGGATGTAA
GCLC [NM_001498.3]	AGGTGACACTATAGAATA ATGAAGCAATAAACAAGCAC	GTACGACTCACTATAGGG ATGGAATGTCACCTGGAG
GST [NM_015917.2]	AGGTGACACTATAGAATA ATACATGGCAAATGACTTAAA	GTACGACTCACTATAGGG ATGATGTCTTCATTCCTTGAC
HO-1 [NM_002133.2]	AGGTGACACTATAGAATA ACTGCGTTCCTGCTCAACAT	GTACGACTCACTATAGGG AGGGCAGAATCTTGCACTTTGT
IκB [NM_020529.2]	AGGTGACACTATAGAATA CTGCAGCAGACTCCAC	GTACGACTCACTATAGGG AGGGTATTTCCTCGAAAGT
JNK [NM_001323327.1]	AGGTGACACTATAGAATA AAGGAAAACGTGGATTTATG	GTACGACTCACTATAGGG ACCAGCATATTTAGGTCTGTT
MAPT [NM_001123066.3]	AGGTGACACTATAGAATA CCCAGATCTGAGAGAGGT	GTACGACTCACTATAGGG ACTTATTAATTATCTGCACCTTCC
MKP1 [NM_004417.3]	AGGTGACACTATAGAATA AGAAGAACCAAATACCTCAA	GTACGACTCACTATAGGG ACAGGTCATAAATAATCAGCA
NF-κB [NM_002908.3]	AGGTGACACTATAGAATA CGTTTTAGATACAAATGTGAAG	GTACGACTCACTATAGGG ACACTTTTCCTTTTCCATAAT
NQO1 [NM_000903.2]	AGGTGACACTATAGAATA CTGCGAACTTTCAGTATCC	GTACGACTCACTATAGGG AGAAGGGTCCTTTGTCATAC
Nrf2 [NM_006164.4]	AGGTGACACTATAGAATA TCGCAAACAACTCTTTATCT	GTACGACTCACTATAGGG AAGAGGAGGTCTCCGTTA
p38 [NM_001315.2]	AGGTGACACTATAGAATA TGAGCTGAAGATTCTGGA	GTACGACTCACTATAGGG ATGTCAGACGCATAATCTG
PP5 [NM_006247.3]	AGGTGACACTATAGAATA CAAGGACTACGAGAACGCCA	GTACGACTCACTATAGGGA GCTTCACCTTGACCACCGTC
PP2A [NM_002715.3]	AGGTGACACTATAGAATA CCGCCATTACAGAGAG	GTACGACTCACTATAGGGA AGGATTTCTTTAGCCTTCT
SOD1 [NM_000454.4]	AGGTGACACTATAGAATA AAGTACAAAGACAGGAAACG	GTACGACTCACTATAGGGA TGACAAGTTTAATACCCATCT
SOD2 [NM_000636.3]	AGGTGACACTATAGAATA ACAACAGGCCTTATTCC	GTACGACTCACTATAGGGA AGAGCTTAACATACTCAGCA

AKT: serine/threonine protein kinase; APP: amyloid precursor protein; BACE1: β-site amyloid precursor protein cleaving enzyme; GCLC: glutamate-cysteine ligase catalytic; GST: glutathione S transferase; HO-1: heme oxygenase-1; IκB: inhibitory kappa B protein; JNK: C-Jun N-terminal kinase; MAPT: microtubule-associated protein tau; MKP1: mitogen-activated protein kinase phosphatase 1; NF-κB: nuclear factor kappa B; NQO1: NADP quinone oxidoreductase 1; Nrf2: nuclear factor erythroid 2-related factor 2; p38: 38 subunit protein; PP5: protein phosphatase 5; PP2A: serine/threonine protein phosphatase 2A; SOD1: superoxide dismutase 1; SOD2: superoxide dismutase 2.

## Data Availability

Not applicable.
